# Anatomic relations of the median nerve to the ulnar insertion of the brachialis muscle: safety issues and implications for medial approaches to the elbow joint

**DOI:** 10.1007/s00402-021-03753-y

**Published:** 2021-01-23

**Authors:** Davide Cucchi, Francesco Luceri, Alessandra Menon, Lars Peter Müller, Koroush Kabir, Pietro Simone Randelli, Paolo Arrigoni, Kilian Wegmann

**Affiliations:** 1 Department of Orthopaedics and Trauma Surgery, Universitätsklinikum Bonn, Venusberg-Campus 1, 53127 Bonn, Germany; 2 U.O.C. 1° Clinica Ortopedica, ASST Centro Specialistico Ortopedico Traumatologico Gaetano Pini-CTO, Piazza Cardinal Ferrari 1, Milan, 20122 Italy; 3 Laboratory of Applied Biomechanics, Department of Biomedical Sciences for Health, Università degli Studi di Milano, Via Mangiagalli 31, 20133 Milan, Italy; 4 REsearch Center for Adult and Pediatric Rheumatic Diseases (RECAP-RD), Department of Biomedical Sciences for Health, Università degli Studi di Milano, Via Mangiagalli 31, 20133 Milan, Italy; 5 Center for Orthopedic and Trauma Surgery, University Medical Center, Cologne, Kerpener Straße 62, 50937 Cologne, Germany; 6 Faculty of Medicine and University Hospital, University of Cologne, Kerpener Straße 62, 50937 Cologne, Germany

**Keywords:** Elbow surgery, Median nerve, Nerve injury, Brachialis, Coronoid process

## Abstract

**Introduction:**

Preventing nerve injury is critical in elbow surgery. Distal extension of medial approaches, required for coronoid fracture fixation and graft-replacement, may endanger the median nerve. This study aims to describe an easily identifiable and reproducible anatomical landmark to localize the median nerve distal to the joint line and to delineate how its relative position changes with elbow flexion and forearm rotation.

**Materials and methods:**

The median nerve and the ulnar insertion of the brachialis muscle were identified in eleven fresh-frozen cadaveric specimens after dissection over an extended medial approach. The elbow was brought first in full extension and then in 90° flexion, and the shortest distance between the two structures was measured while rotating the forearm in full pronation, neutral position and full supination.

**Results:**

The distance between the median nerve and the brachialis insertion was highest with the elbow flexed and the forearm in neutral position. All distances measured in flexion were larger than those in extension, and all distances measured from the most proximal point of the brachialis insertion were larger than those from the most distal point. Distances in pronation and in supination were smaller than to those in neutral forearm position.

**Conclusions:**

The ulnar insertion of the brachialis is a reliable landmark to localize and protect the median nerve at the level of the coronoid base. Elbow flexion and neutral forearm position increase significantly the safety margins between the two structures; this information suggests some modifications to the previously described medial elbow approaches.

**Level of evidence:**

Basic Science Study.

## Introduction

Detailed knowledge of the anatomical relation between nerves and bony or muscular landmarks is critical in surgical approaches. In that regard, the elbow has a special situation, as three major nerves pass the joint and regularly have to be identified and protected. Fixation of coronoid fractures is known to be a challenging procedure [1–4], as well as graft-replacement of the coronoid [5–8]. To address such pathologies, the Hotchkiss and flexor carpi ulnaris (FCU) splitting approaches are commonly used, besides other approaches to the medial elbow [9–17]. In contrast to ligament procedures on the medial side, for which these approaches can be used as well, fracture fixation, coronoid replacement and revision cases may demand more extensile dissection. In fact, the base of the coronoid, hidden under the distal insertion of the brachial muscle at the ulnar tuberosity, must often be visualized. A delicate task when doing this is a soft tissue dissection respectful of the complex neuroanatomy of the anteromedial aspect of the elbow, devoting special attention to the median nerve. An excellent study by Sukegawa et al. nicely displayed landmarks to easily locate the median nerve with respect to the medial epicondyle [18]. However, to identify the median nerve at the level of the base of the coronoid using Sugekawa’s landmarks, it would be necessary to release the flexor-pronator mass proximally, which is not always necessary. Furthermore, the study did not elaborate on the influence of forearm position on median nerve movements with respect to bony landmarks. To the best of our knowledge, no applicable medial landmarks have been presented, to help in easily identifying the median nerve distal to the base of the coronoid. In contrast to this, to facilitate lateral approaches, many experimental studies recommended safe-zones and reported the precise anatomical course of the radial nerve and its branches in relation to bony and soft-tissue landmarks, taking into account also the role of different elbow and forearm positions [19–24]. For the median nerve, reliable landmarks have scarcely been reported, mostly by studies performed in arthroscopic settings and focused on defining anatomical relations at the level of the joint line, without investigating more distal regions. [13, 17, 18, 25–29]. Nevertheless, knowledge of median nerve position and behavior is essential in complex open surgery and revision cases and constitutes a precious help for all surgeons dealing with medial approaches, especially if lacking in a dedicated subspecialistic training on elbow surgery.

The aim of the present study was to fill this gap in surgical anatomy knowledge and to investigate how elbow flexion and forearm movements change the relative position of the median nerve distally to the elbow joint line, with special attention to the anatomical relations between the median nerve and the ulnar insertion of the brachialis muscle, to present a reproducible landmark.

## Materials and methods

**Fig. 1:**
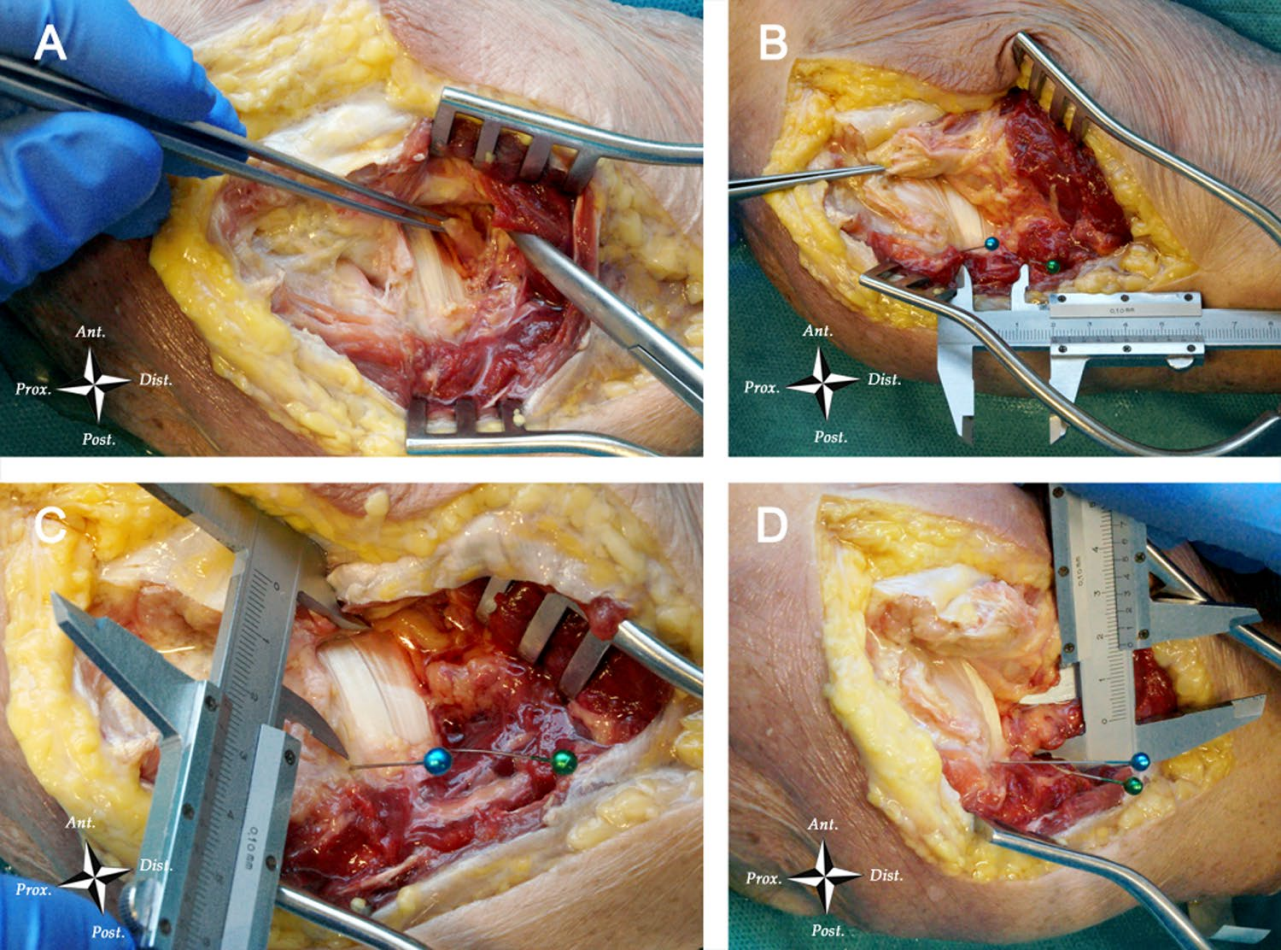
Surgical dissection of the medial aspect of the elbow. **a** Identification of the median nerve. **b** Identification of the most proximal (blue pin) and most distal (green pin) points of the ulnar insertion of the brachialis muscle and measurement of the “brachialis insertion length” with a graduated caliper. **c** Measurement of the shortest linear distance between the median nerve and the most proximal point of the distal end of the brachialis (blue pin) with a graduated caliper. **d** Measurement of the shortest linear distance between the median nerve and the most distal point of the distal end of the brachialis (green pin) with a graduated caliper. *Ant.* Anterior, *Post.* Posterior, *Dist.* distal, *Prox.* proximal

Eleven fresh-frozen cadaveric specimens (including the middle third of the humerus and the entire forearm and hand) without pathologies or traumatic injuries to the bony and nervous structures of the elbow were dissected using an extended medial approach. After subcutaneous dissection, the fascia was incised sharply from 20 mm proximal to the flexor-pronator origin to the mid-point of the medial aspect of the ulna and was elevated in anterolateral direction; subsequently, the entire flexor-pronator mass was detached from the medial epicondyle, lifted off from the capsule and reflected anterolaterally. The ulnar head of the pronator teres was then exposed and removed, revealing the ulnar insertion of the brachialis. The median nerve was then identified lying on the tendinous portion or on the muscle belly of the brachialis; the brachial artery and veins were not dissected to avoid excessive tissue mobilization (Fig. 1a).

The most proximal and most distal points of the brachialis insertion on the ulna were marked for subsequent measurements, and the distance between these two points was measured with a graduated caliper and defined as “brachialis insertion length” (Fig. 1b).

Subsequently, a graduated caliper was used to measure the shortest linear distance between the median nerve and the previously marked proximal and distal ends of the ulnar insertion of the brachialis muscle (Fig. 1c, d). These two measurements were repeated with the elbow in 90° flexion with the forearm in full pronation, neutral position and full supination and with the elbow in full extension, with the forearm in full pronation, neutral position and full supination. All measurements were then normalized to the specimen’s transepicondylar distance (ratio between distance measured and transepicondylar distance) [30]. Two examiners performed all measurements simultaneously reaching mutual agreement on each passage (D.C. and F.L.).

Institutional approval of the study protocol was obtained (ID 19931—Nicola’s Foundation & ICLO Research Center).

Statistical analysis (A.M.) was performed using GraphPad Prism v 6.0 software (GraphPad Software Inc.). The normal distribution of the sample was evaluated with the Shapiro–Wilk normality test. Continuous variables were expressed as median and interquartile range (first and third quartiles) or as mean and standard deviation, as appropriate. After analysis of outliers, statistical evaluation of the differences among the groups was performed using repeated measures one-way analysis of variance (ANOVA) with post hoc Tukey’s multiple comparisons test. The significance level was set at *p* value lower than 0.05.

## Results

The median nerve could be identified in all eleven specimens [females: 63.6%; age at death: 73 (± 12.9) years; left elbow: 54.5%; transepicondylar distance 60.3 (± 4.4) mm]. The mean brachialis insertion length was 27.9 (± 2.1) mm.

The mean (and normalized) distances between the nerve and the brachialis muscle in the different forearm positions are reported in Table 1. The maximum distance was obtained from the most proximal point of the brachialis insertion in flexion and neutral forearm position, whereas the minimal distance was obtained from the most distal point of the brachialis insertion in extension and forearm pronation.

**Table 1:** Absolute and normalized distances between the nerve and the musculus brachialis obtained in the different study conditions

	Testing condition		Distance between the nerve and the musculus brachialis	
	Elbow position	Forearm rotation	Absolute (mm)	Normalized to the TED (%)
Proximal	Extension	Pronation	11.0 [9.0–12.0]	18.6 (± 4.4)
		Neutral	13.3 (± 2.3)	22.1 (± 4.6)
		Supination	10.9 (± 2.7)	18.3 (± 4.9)
	Flexion	Pronation	22.0 [21.5–24.0]	36.0 (± 7.3)
		Neutral	28.0 [25.5–29.5]	44.2 (± 7.2)
		Supination	24.0 [20.0–25.5]	37.1 (± 8.2)
Distal	Extension	Pronation	2.8 (± 2.7)	4.6 (± 4.3)
		Neutral	6.4 (± 2.5)	10.6 (± 4.0)
		Supination	4.0 (± 1.7)	6.6 (± 2.9)
	Flexion	Pronation	9.8 (± 3.7)	16.2 (± 6.2)
		Neutral	14.0 [11.0–15.0]	20.6 (± 6.2)
		Supination	10.4 (± 3.1)	17.2 (± 5.0)

Data are reported as mean (± SD) or median [Q1–Q3]

*Q1* first quartile, *Q3* third quartile, *SD* standard deviation, TED transepicondylar distance

Subsequently, repeated measures one-way ANOVA was performed on subsets of measurements with the same degree of elbow flexion. A significant interaction was documented for the distances measured from both the proximal and distal points and in all elbow flexion grades (proximal, flexion: *p* < 0.0001; proximal, extension: *p* = 0.0027; distal, flexion: *p* < 0.0001; distal, extension: *p* = 0.0006).

**Fig. 2:**
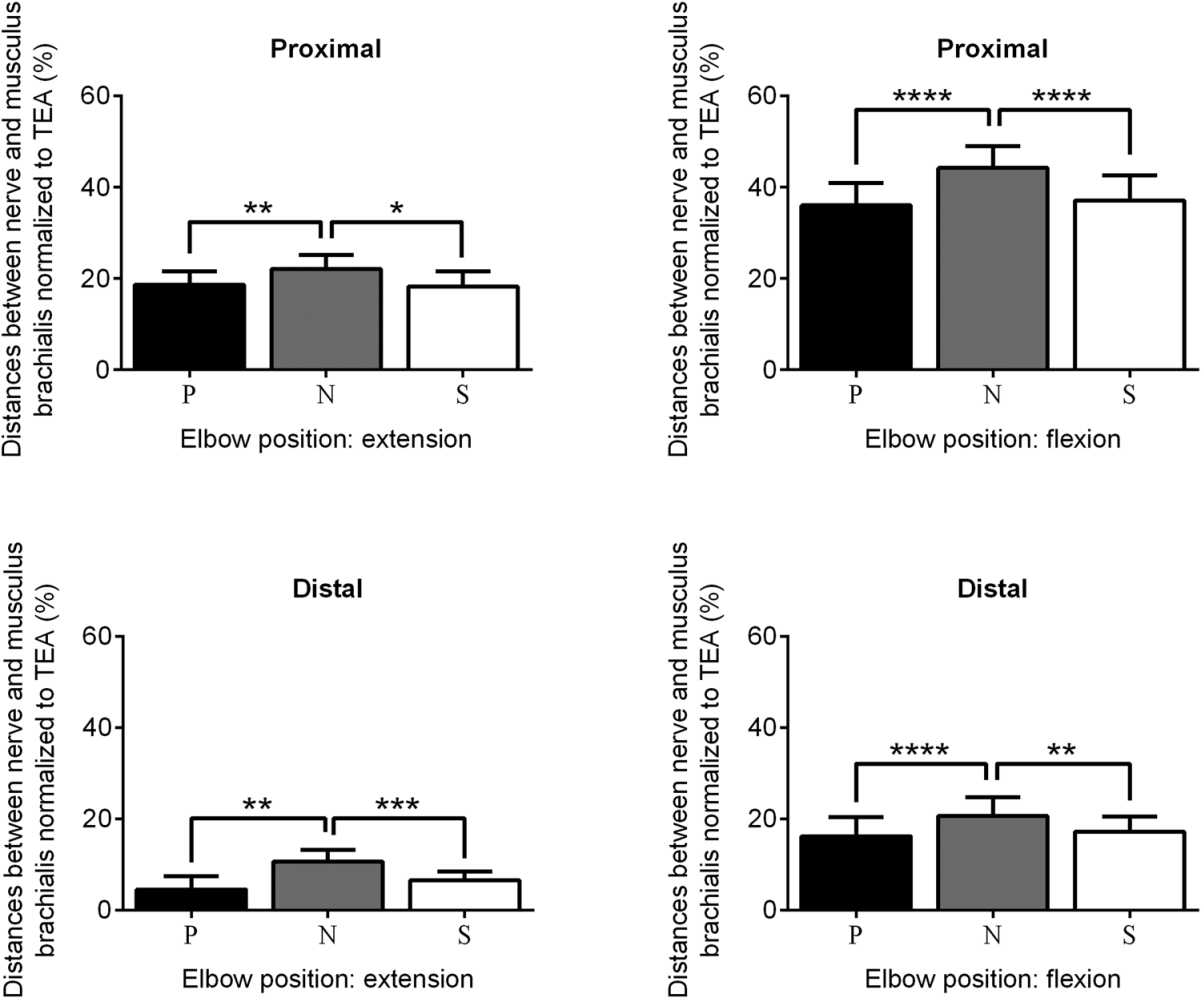
Comparison of distances between median nerve and ulnar insertion of the brachialis muscle obtained in different study conditions, highlighting the role of forearm movements. Each box represents the mean distance normalized to the TED. The error bars show the 95% confidence interval values. A Tukey’s multiple comparisons test was used to test for differences between measurements obtained from the same point of the brachialis insertion and at the same degree of elbow flexion, differing between each other only for forearm rotation. *N* neutral position, *P* pronation, *S* supination, *TED* transepicondylar distance. Only *p* values < 0.05 are indicated: *p < 0.05; **p < 0.01; ***p < 0.001; ****p < 0.0001

The results of the Tukey’s multiple comparisons test revealed statistically significant differences when comparing measurements in pronation or supination to those in neutral position, but not when comparing measurements obtained in pronation with those in supination (Table 2, Fig. 2).

**Table 2:** Summary of the results of the Tukey’s multiple comparisons test when comparing the different study conditions

	Elbow position	Comparison	Mean difference (%; 95% CI)	*p* value
Proximal	Extension	Pronation–Neutral	− 3.5 (− 5.7 to − 1.2)	0.0052
		Pronation–Supination	0.4 (− 2.6 to 3.3)	n.s.
		Neutral–Supination	3.8 (0.8 to 6.8)	0.0153
	Flexion	Pronation–Neutral	− 8.2 (− 10.0 to − 6.3)	< 0.0001
		Pronation–Supination	− 1.1 (− 3.3 to 1.1)	n.s.
		Neutral–Supination	7.1 (4.8 to 9.4)	< 0.0001
Distal	Extension	Pronation–Neutral	− 6.1 (− 9.8 to − 2.3)	0.0031
		Pronation–Supination	− 2.0 (− 5.2 to 1.2)	n.s.
		Neutral–Supination	4.1 (2.1 to 6.1)	0.0007
	Flexion	Pronation–Neutral	− 4.4 (− 6.0 to − 2.7)	< 0.0001
		Pronation–Supination	− 1.0 (− 3.1 to 1.1)	n.s.
		Neutral–Supination	3.4 (1.3 to 5.5)	0.0035

Data are reported as mean and 95% confidence interval (CI)

*n.s.* not significant

When comparing pairs of measures obtained at the same degree of forearm rotation, all distances measured in flexion appeared significantly larger than their counterparts measured in extension (proximal, pronation: *p* < 0.0001; proximal, neutral: *p* < 0.0001; proximal, supination: *p* < 0.0001; distal, pronation: *p* = 0.0001; distal, neutral: *p* = 0.0002; distal, supination: *p* < 0.0001).

**Fig. 3:**
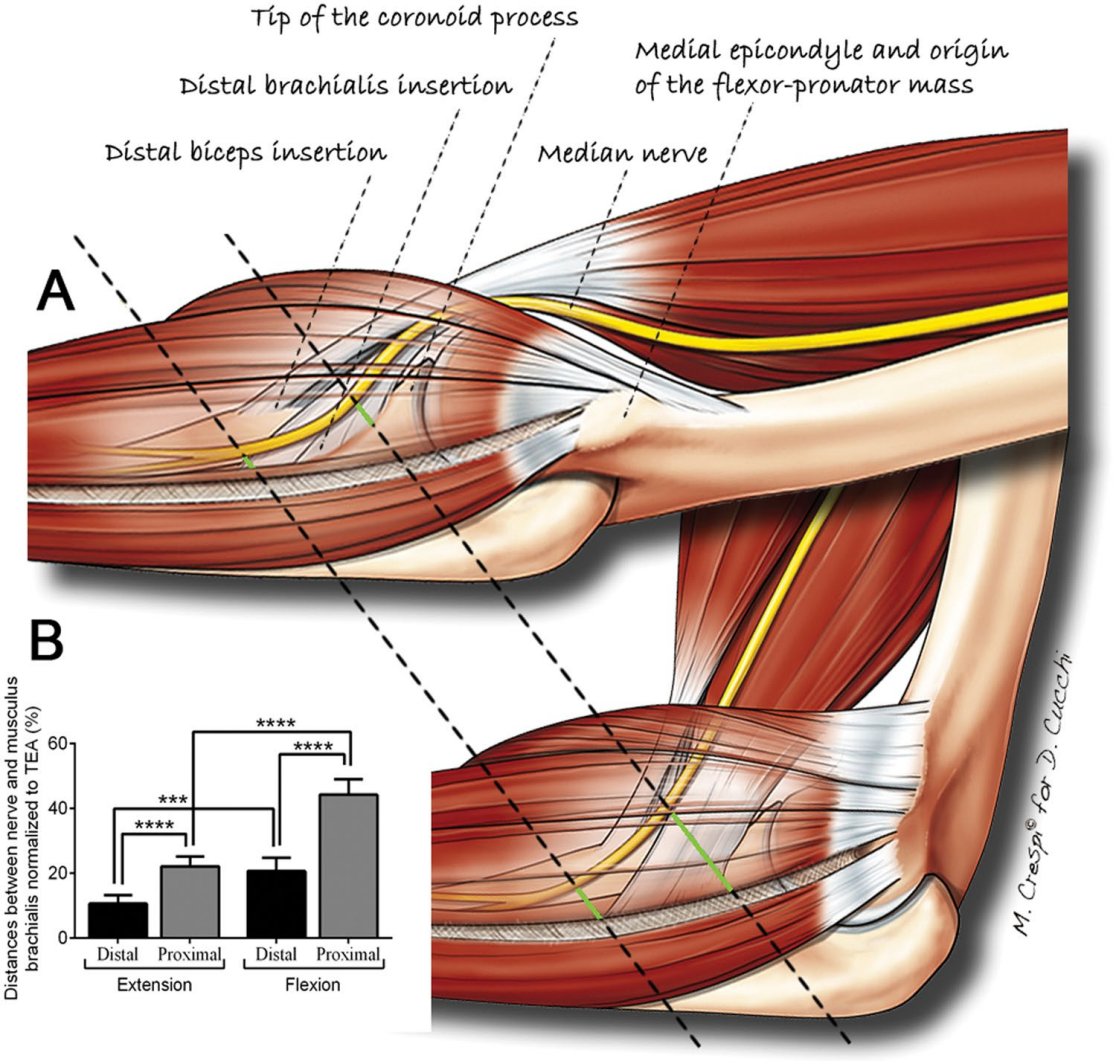
Summary of the main study results. **a** Diagram of the medial aspect of the elbow in full extension and 90° flexion with two superimposed black dashed lines, representing the measurement direction. The green segments highlight the distance between the median nerve and the most proximal and the most distal points of the brachialis insertion in different study conditions. **b** Comparison of distances between median nerve and the brachialis muscle obtained in different study conditions, highlighting the role of the measurement point on the brachialis insertion and of elbow flexion. Each box represents the mean distance normalized to the TED. The error bars show the 95% confidence interval values. A Tukey’s multiple comparisons test was used to test for differences between measurements obtained from different points of the brachialis insertion and at different degrees of elbow flexion. All illustrated measurements were conducted with the forearm in neutral position. *TED* transepicondylar distance. Only *p* values < 0.05 are indicated: *p < 0.05; **p < 0.01; ***p < 0.001; ****p < 0.0001

When comparing pairs of measures obtained from the most proximal and most distal points of the brachialis insertion with identical conditions of elbow flexion and forearm rotation, all distances measured from the most proximal point of the brachialis insertion appeared significantly larger than their counterparts measured from the most distal point of the brachialis insertion (*p* < 0.0001 for all comparisons). Figure 3 summarizes the main study results.

## Discussion

The main finding of this study is that the relative position of the median nerve to the ulnar insertion of the brachialis muscle changes with different grades of elbow flexion and forearm movements. In particular, this study demonstrated that the distance between the median nerve and the ulnar insertion of the brachialis muscle is maximal with the elbow flexed and the forearm in neutral position and significantly decreases with elbow extension, irrespective of pronation and supination. Also, we defined the ulnar insertion of the brachialis muscle as a reliable landmark in identifying the position of the median nerve during surgical dissection of the medial aspect of the elbow.

The median nerve (Cervical 5 to Thoracic 1) originates from the medial and lateral cords of the brachial plexus. In the arm, the nerve is contained in a fascial sheath continuous with the fascia of the brachialis and biceps muscles and runs in close proximity with the brachial artery, remaining medial to the brachialis muscle [13]. At the level of the distal humerus, nerve and artery lie in a groove between the brachialis and the biceps brachii bellies and enter the antecubital fossa in anterolateral direction. Here the nerve remains medial to the brachial artery and to the biceps brachii tendon and underneath the bicipital aponeurosis, being located ventral to the medial quarter of the humeral trochlea on the coronal plane [16]. After separating from the brachial artery, which sinks into the antecubital fossa and divides in the radial and the ulnar branches approximately at the level of the coronoid process, the nerve passes in the forearm between the humeral and ulnar head of the pronator teres muscle [28]. Rare variations of the course of the median nerve associated with anatomical variations of the distal humerus, the musculocutaneus nerve and the brachial artery have been encountered and described [1, 7, 13, 24].

The knowledge of the close relation of the median nerve with easily identifiable muscular structures of the anteromedial aspect of the elbow is relevant in all open approaches to this area. Several approaches to the medial and anteromedial structures of the elbow have been proposed [9], including numerous variations of the FCU splitting initially proposed by Jobe [10–12], the Hotchkiss “over the top” approach [13], and the rarely used transepicondylar and posteromedial approaches described by Molesworth [14] and Campbell [15] and by Taylor and Scham [16].

A critical point of the medial approaches to the elbow is the need to respect the nerval structures, especially the median nerve. The over the top approach was initially described for contracture releases and is frequently used also to treat coronoid fractures, since it provides a favorable field of view of the anteromedial elbow joint respecting the internervous plane between the flexor-pronator mass, which is innervated by the median nerve, and the FCU, innervated by the ulnar nerve [13]; however, a more distal extension of this approach raises safety issues, since it may endanger the median nerve. As opposed to this approach, the FCU splitting approach offers a superior osseous exposure of the coronoid process, enabling exposure of all potential fracture subtypes of the anteromedial coronoid fractures [31]. This approach is considered technically easier and less invasive than the over the top approach; however, it is not an internervous approach; therefore, an excessive extension may put the innervation of either head of the FCU at risk, if the “safe area” for the muscle split (up to 10 mm distal to the sublime tubercule [10]) is not carefully respected. Furthermore, it forces the surgeon to move the ulnar nerve out of its bed, potentially leading to scarring around the nerve. The importance of nerve protection in distal elbow exposures has encouraged the development of alternative approaches, which permit to identify distal structures without endangering nerve structures [17].

In all approaches, nerve protection during exposure is a critical issue; however, as opposed to lateral ones, only few studies investigated how changes in forearm position affect the relative position of the median nerve with respect to medial landmarks. The present study was conducted with the aim to describe, regardless of the surgical approach used, the anatomical relation of the course of the median nerve in relation to a reproducible medial landmark, and to delineate how the nerve position changes with respect to that landmark, therefore updating previous studies, which described this relationship in a static fashion [18]. This detailed anatomical description and the information that the median nerve becomes more distant from the ulnar insertion of the brachialis muscle with the elbow flexed and the forearm in neutral position is precious for elbow surgeons, especially for young ones and those without a dedicated subspecialistic training in elbow surgery, for whom medial elbow approaches can be extremely challenging. Furthermore, the results of this study suggest modifications to the previously described medial approaches, encouraging operating with the elbow at higher degrees of flexion. In fact, most of the approaches are currently performed with the elbow positioned at 30° flexion and the medial epicondyle facing toward the surgeon, and with this setting are also conducted the available anatomical studies describing nerve position [13, 17, 18].

The role of elbow flexion in changing the nerve distances to bony landmarks has been extensively investigated in relation to portal placement for elbow arthroscopy. Hackl et al. demonstrated that the distance of the median nerve to the anterior tip of the coronoid and to the anterior border of the trochlea significantly increases from extension to 90° flexion [25]. These findings were supported by a recent review of cadaveric studies, which concluded that the distended elbow in a 90° flexed position minimizes the risk for neurovascular injury with the arthroscope [26].

This study extended the validity of these results obtained with an arthroscopic setting to open surgery, providing relevant information for surgeons performing procedures close to the ulnar insertion of the brachialis, such as open reduction and internal fixation of coronoid fractures with plates, especially if screws placed in anteroposterior direction are required [1–4, 32], or coronoid replacement with a graft [5–8].

The role of forearm rotation was also mainly studied in relation to the placement of arthroscopic portals. Conflicting results were published, with studies suggesting that the distance of the median nerve to the anteromedial portal can be increased by forearm supination [27], others by pronation [28], and others not being able to show any influence of forearm rotation [25].

These previous studies were focused on an arthroscopic setting, and all evaluated the relative position of landmarks to the median nerve at the level of the elbow joint line. Here, the soft tissues in the antecubital fossa become progressively more mobile with increasing flexion [33–35]. Therefore, it is reasonable to expect an increased mobility of the nerve at the level of the joint line in 90° flexion, which simulates well elbow arthroscopy. However, this behavior was not yet investigated distally to this level, which may have greater relevance for open approaches to a fractured coronoid process.

In a trauma surgery setting, the bony profile of the anterior ulna, the elbow joint capsule and the medial collateral ligamentous complex might be damaged from the trauma, leaving the medial epicondyle and the ulnar shaft as only reliable references. Here, the distal insertion of the brachialis muscle is considered as a precious and reproducible landmark, of particular relevance for surgeons not routinely dealing with medial elbow approaches, such as frequently occurs in traumatology departments. The ulnar insertion varies in shape between individuals, with an average length ranging between 21 and 44 mm across different studies [36, 37]. The measurements of the brachialis insertion length we obtained fall between the ranges of previously published studies, confirming the reliability of this structure as a reproducible landmark.

Isolated injury to the brachialis is uncommon, but lesions to the proximal part of the ulnar insertion may occur in combination with anteromedial coronoid fractures [32, 38]: therefore, detecting Regan and Morrey type III fracture on radiographs should raise awareness for brachialis tendon injuries, triggering second level diagnostics, such as magnetic resonance imaging, and advising care when approaching this region surgically [38].

This study has some limitations. The age of patients who usually undergo elbow surgery is lower than that of the included specimens, which suggests care when transferring these results to clinical practice. However, ageing is not supposed to significantly influence nerve position and behavior. Secondly, the studied population is relatively small: this could amplify bias related to anatomical variants and to the dissection technique, although this was performed meticulously and kept to a minimum. Finally, we focused primarily on the description of the position of the median nerve in relation to the brachialis muscle. The study was neither designed to investigate the risk of nerve injuries in specific surgical procedures, nor to detect the effect of systemic connective tissue diseases or local pathological changes on the soft tissue of the medial side of the elbow.

## Conclusions

Nerve protection during surgical exposure around the elbow is a critical issue. This anatomical study demonstrated that the relative position of the median nerve to the ulnar insertion of the brachialis muscle is maximal with the elbow flexed and the forearm in neutral position and significantly decreases with elbow extension and both with forearm pronation and supination. This information can be precious to elbow surgeons, suggesting some modifications to the previously described medial elbow approaches.

## References

[1] Morellato J, Louati H, Desloges W (2018). Fixation of anteromedial coronoid facet fractures. J Orthop Trauma.

[2] Rashid A, Copas D, Granville-Chapman J, Watts A (2019). Arthroscopically-assisted fixation of anteromedial coronoid facet fracture and lateral ulnar collateral ligament repair for acute posteromedial rotatory fracture dislocation of the elbow. Shoulder Elb.

[3] Rausch V, Hackl M, Seybold D (2020). Plattenosteosynthese des Processus coronoideus ulnae. Oper Orthop Traumatol.

[4] Shen J-J, Qiu Q-M, Gao Y-B (2019). Direct anterior approach for mini plate fixation of Regan-Morrey type II comminuted ulnar coronoid process fracture. J Orthop Surg (Hong Kong).

[5] Chung C-H, Wang S-J, Chang Y-C, Wu S-S (2007). Reconstruction of the coronoid process with iliac crest bone graft in complex fracture-dislocation of elbow. Arch Orthop Trauma Surg.

[6] Silveira GH, Bain GI, Eng K (2013). Reconstruction of coronoid process using costochondral graft in a case of chronic posteromedial rotatory instability of the elbow. J shoulder Elb Surg.

[7] Bellato E, Rotini R, Marinelli A (2016). Coronoid reconstruction with an osteochondral radial head graft. J shoulder Elb Surg.

[8] van Riet RP, Morrey BF, O’Driscoll SW (2005). Use of osteochondral bone graft in coronoid fractures. J Shoulder Elb Surg.

[9] Patterson SD, Bain GI, Mehta JA (2000). Surgical approaches to the elbow. Clin Orthop Relat Res.

[10] Smith GR, Altchek DW, Pagnani MJ, Keeley JR (1996). A muscle-splitting approach to the ulnar collateral ligament of the elbow. Neuroanatomy and operative technique. Am J Sports Med.

[11] Ring D, Doornberg JN (2007). Fracture of the anteromedial facet of the coronoid process. J Bone Jt Surg.

[12] Jobe FW, Stark H, Lombardo SJ (1986). Reconstruction of the ulnar collateral ligament in athletes. J Bone Jt Surg Am.

[13] Hotchkiss RN, Kasparyan GN (2000). The medial “over the top” approach to the elbow. Tech Orthop.

[14] Molesworth HWL (1930). An operation for the complete exposure of the elbow-joint. Br J Surg.

[15] Campbell WC (1932). Incision for exposure of the elbow joint. Am J Surg.

[16] Taylor TK, Scham SM (1969). A posteromedial approach to the proximal end of the ulna for the internal fixation of olecranon fractures. J Trauma.

[17] Jost B, Benninger E, Erhardt JB (2015). The extended medial elbow approach—a cadaveric study. J Shoulder Elb Surg.

[18] Sukegawa K, Suzuki T, Ogawa Y (2016). Anatomical cadaver study of the hotchkiss over-the-top approach for exposing the anteromedial facet of the ulnar coronoid process: critical measurements and implications for protecting the median nerve. J Hand Surg Am.

[19] Hackl M, Wegmann K, Lappen S (2015). The course of the posterior interosseous nerve in relation to the proximal radius: is there a reliable landmark?. Injury.

[20] Calfee RP, Wilson JM, Wong AHW (2011). Variations in the anatomic relations of the posterior interosseous nerve associated with proximal forearm trauma. J Bone Jt Surg Am.

[21] Diliberti T, Botte MJ, Abrams RA (2000). Anatomical considerations regarding the posterior interosseous nerve during posterolateral approaches to the proximal part of the radius. J Bone Jt Surg Am.

[22] Tornetta P, Hochwald N, Bono C, Grossman M (1997). Anatomy of the posterior interosseous nerve in relation to fixation of the radial head. Clin Orthop Relat Res.

[23] Arrigoni P, Cucchi D, Menon A (2019). The posterior interosseous nerve crosses the radial head midline and increases its distance from bony structures with supination of the forearm. J Shoulder Elb Surg.

[24] Cucchi D, Arrigoni P, Luceri F (2019). Modified anteromedial and anterolateral elbow arthroscopy portals show superiority to standard portals in guiding arthroscopic radial head screw fixation. Knee Surgery, Sport Traumatol Arthrosc.

[25] Hackl M, Lappen S, Burkhart KJ (2015). Elbow positioning and joint insufflation substantially influence median and radial nerve locations. Clin Orthop Relat Res.

[26] Cushing T, Finley Z, O’Brien MJ (2019). Safety of anteromedial portals in elbow arthroscopy: a systematic review of cadaveric studies. Arthrosc J Arthrosc Relat Surg.

[27] Drescher H, Schwering L, Jerosch J, Herzig M (2008). Das Risiko Neurovaskulärer Schädigungen bei der Ellenbogengelenkarthroskopie. Z Orthop Ihre Grenzgeb.

[28] Unlu MC, Kesmezacar H, Akgun I (2006). Anatomic relationship between elbow arthroscopy portals and neurovascular structures in different elbow and forearm positions. J shoulder Elb Surg.

[29] Arrigoni P, Cucchi D, Guerra E (2019). No neurovascular damage after creation of an accessory anteromedial portal for arthroscopic reduction and fixation of coronoid fractures. Knee Surg Sports Traumatol Arthrosc.

[30] Kamineni S, Ankem H, Patten DK (2009). Anatomic relationship of the radial nerve to the elbow joint: clinical implications of safe pin placement. Clin Anat.

[31] Huh J, Krueger CA, Medvecky MJ, Hsu JR (2013). Medial elbow exposure for coronoid fractures. J Orthop Trauma.

[32] Ma J-F, Chang S-M (2011). Brachialis insertion measurement: an anatomic cadaver study for plate fixation of the coronoid process fracture. Clin Anat.

[33] King GJ, Morrey BF, An KN (1993). Stabilizers of the elbow. J shoulder Elb Surg.

[34] Malagelada F, Dalmau-Pastor M, Vega J, Golanó P, Doral MN, Karlsson J (2014). Elbow anatomy. Sport. Inj.

[35] de Haan J, Schep NWL, Eygendaal D (2011). Stability of the elbow joint: relevant anatomy and clinical implications of in vitro biomechanical studies. Open Orthop J.

[36] Kamineni S, Bachoura A, Behrens W (2015). Distal insertional footprint of the brachialis muscle: 3D morphometric study. Anat Res Int.

[37] Leonello DT, Galley IJ, Bain GI, Carter CD (2007). Brachialis muscle anatomy. J Bone Jt Surg.

[38] Sanal HT, Chen L, Negrao P (2009). Distal attachment of the brachialis muscle: anatomic and MRI study in cadavers. Am J Roentgenol.

